# The clinical application of venous ultrasound in diagnosis and follow-up of lower extremity deep vein thrombosis (DVT): a case-based discussion

**DOI:** 10.1186/s12959-023-00550-y

**Published:** 2023-10-26

**Authors:** Farooq Akram, Bingwen Eugene Fan, Chuen Wen Tan, Wey Chyi Teoh, Paolo Prandoni, Eng Soo Yap

**Affiliations:** 1 Department of Medicine, Changi General Hospital, Singapore, Singapore; 2 DUKE NUS School of Medicine, Singapore, Singapore; 3 Lee Kong Chian School of Medicine, Nanyang Technological University, Singapore, Singapore; 4 Yong Loo Lin School of Medicine, National University of Singapore, Singapore, Singapore; 5 Department of Haematology, Tan Tock Seng Hospital, Novena, Singapore; 6 Department of Laboratory Medicine, Khoo Teck Puat Hospital, Yishun, Singapore; 7 Department of Hematology, Singapore General Hospital, Singapore, Singapore; 8 Department of Diagnostic Radiology, Changi General Hospital, Simei, Singapore; 9 Arianna Foundation on Anticoagulation, Bologna, Italy; 10 Department of Laboratory Medicine, National University Hospital, Singapore, Singapore

**Keywords:** DVT, Venous ultrasound, Follow-up

## Abstract

Current clinical practice guidelines lack explicit guidance on the indications and appropriate timing of venous ultrasound (US) in lower extremity deep vein thrombosis (DVT) follow-up. Moreover, abnormal findings reported on venous US in DVT follow-up or suspected recurrent DVT may be difficult for clinicians to interpret, which carries risk of harm from inappropriate use of anti- coagulation and increased healthcare resource utilization. Due to the above factors, over-use of ultrasound in diagnosis and follow-up of lower extremity DVT has been reported in western health systems. We have undertaken a case-based discussion and a scoping review of existing guidelines on the use of venousUS following prior diagnosis of DVT, to guide appropriate interpretation of commonly reported US abnormalities and provide our suggestions in the light of best available evidence on appropriate timing to perform follow-up US in management of lower extremity DVT.

## Background

Complete duplex ultrasound (CDUS) is the preferred venous ultrasound protocol for diagnosis of acute DVT, with pooled sensitivity and specificity to be 94% and 97.3% respectively, which involves compression of deep veins from inguinal ligament to the ankle and doppler waveforms in common femoral and popliteal vein [1]. Despite being an excellent diagnostic modality, appropriate patient selection is crucial for improving diagnostic accuracy and appropriate interpretation of US abnormalities. Due to increasing awareness, suspicion of venous thromboembolism (VTE) has increased which has lowered the threshold to order venous US. However, only 20% of suspected cases have a confirmed diagnosis of VTE [2].

Previous studies have shown inappropriate use of ultrasound scans in DVT acute diagnosis and follow-up. A study by Fowl et al. on 2993 lower extremity venous duplex scans performed to diagnose acute DVT, surveillance in high risk groups and follow-up of previously diagnosed DVT, concluded there was over-use of ultrasound scans due to a very high percentage of normal results [3].

Meissner reviewed the significance of repeat duplex US in DVT follow-up and concluded that routine ultrasound follow-up in proximal DVT was unwarranted, except for an end of therapy ultrasound in those at high risk of future recurrence [4].

A retrospective study noted high incidence of inappropriate routine use of compression ultrasound scans in lower extremity cellulitis. The incidence of DVT was 8% (11/133 compression ultrasound scans performed) while 8/11 were previously diagnosed with DVT and only 3 were new [5].

In our practice, similar to previous studies [3–5], some of the questionable venous US requests observed were to diagnose DVT in confirmed acute cellulitis, persistent but improved leg swelling following DVT at clinic follow-up while on anti-coagulation, and symmetrical bilateral leg swelling. Another example would be in the event of contraindication to anti-coagulation arising during extended phase of DVT treatment without new leg symptoms: to determine the need for IVC filter (if thrombus present) or discontinuation of anti-coagulation (in the case of recanalization).

Lastly, frequent US requests following acute DVT have been observed, which were performed mainly due to the common misunderstanding that complete or significant deep vein recanalization is a safe marker to stop anticoagulation. This has become the basis for repeating one or even several CDUS to make decisions whether to stop or extend anticoagulation. This practice is controversial, expensive and poses risk of harm from unwarranted anticoagulation continuation or premature discontinuation, use of invasive procedures such as inferior vena cava (IVC) filter and patient inconvenience while stretching the available healthcare resources. We have identified two key factors which contribute to potential inappropriate use of venous US. Firstly, clinicians may find difficulty in interpretation of US abnormal findings due to knowledge gaps and discordant literature reporting. Moreover, lack of standardized terminology, subjectivity and inter-reporter variability in US reporting are additional factors which may lead to potential misunderstanding among clinicians.

Secondly, only a few VTE guidelines briefly describe the role of venous US in DVT follow-up. Some of the guidelines suggest an end of anticoagulation therapy ultrasound (US), but fail to provide guidance on appropriate timing to perform US.

We would like to present this review in two sections, keeping some of the above mentioned questionable US request observations in mind. Section I will comprise 5 hypothetical case-based discussions related to the diagnosis and follow-up of DVT with venous US, by addressing common US reported findings, which may raise the potential of misinterpretation by clinicians and ultimately may lead to inappropriate use of subsequent ultrasound procedures and over or under anticoagulant treatment. Section II will include a suggested guide on appropriate timing to perform follow-up venous US, to promote evidence based use of US in DVT management and potential cost saving.

## Section 1. Case-based discussion

### Case 1: Sub-acute DVT on CDUS.

A 50-year-old Chinese gentleman with history of chronic bilateral leg swelling due to chronic venous insufficiency (CVI) presented with left leg swelling, which he first noticed 10 weeks ago, after his hospital discharge for pneumonia which was managed in intensive care unit (ICU). He reported significant improvement in left leg swelling 2 weeks before his clinical review. On examination, left calf girth was 1.5 cm more than the right calf. CDUS was performed, which showed a 3 cm long thrombus in the left peroneal vein, which was reported as sub-acute with a recommendation to correlate clinically.

How should we interpret CDUS findings? Is there an indication for anticoagulation?

#### Discussion

In our opinion, the patient likely developed distal or below knee DVT either during or soon after the hospital discharge 10 weeks ago. We attributed this DVT as provoked due to his hospital stay > 3 days and contributed by severe illness.

Though, it is likely that this patient’s DVT was recent onset based on the history provided and documentation from his last admission, however, often there are cases when it is difficult to estimate the onset of DVT due to multiple factors such as absence of reliable history, presence of chronic leg swelling or varying DVT signs and symptoms between patients and prevalence of sub-clinical DVT i.e., which is often discovered incidentally or during investigation of pulmonary embolism. In such circumstances, determining the age of the thrombus based on US alone may be difficult unless clear features of acute or chronic DVT are present.

We sometimes see sub-acute thrombus reports on US which refers to appearance of thrombus which is neither acute nor chronic. In general, a thrombus which is a few weeks to less than six months old may be considered sub-acute [6], but its definition remains rather vague as no time based definition has been described in the literature to provide guidance in differentiating DVT into acute, sub-acute or chronic. However, it should be emphasized that US report of acute or subacute DVT should always be correlated clinically, as sub-acute may be misinterpreted for acute and vice versa, i.e., a patient who developed DVT weeks ago (sub-acute) may be misinterpreted as an acute DVT who did not seek medical attention i.e., no US performed earlier at the time of symptom onset.

The Society of Radiologists in Ultrasound (SRU) in their consensus report 2018 [6] discourage the use of terms such a sub-acute thrombus. The SRU has suggested to classify venous US abnormalities into acute venous thrombosis, chronic post-thrombotic change and indeterminate: when findings are neither completely acute nor chronic.

Acute thrombus usually appears as expanded and hypoechoic, with an increase in venous diameter of the affected segment. The thrombus may still be deformable. Post thrombotic changes tend to be more echogenic, has associated decreased venous diameter, is more rigid and have irregular surfaces. They may have internal synechiae or calcifications [6].

The term sub-acute thrombus may be used only as a follow-up when a recent baseline US is available for comparison, otherwise thrombus may be regarded as indeterminate.

In essence, accurate dating of a subacute thrombus solely on venous US is technically challenging as its appearance can overlap with acute thrombus or chronic post thrombotic change. Magnetic resonance direct thrombus imaging (MRDTI) may differentiate acute thrombus from chronic thrombus, however its utility may be limited by availability and cost [7].

We suggest clinicians to discuss with radiology colleagues if US reports thrombosis without specifying chronicity of the thrombus as acute, indeterminate or post-thrombotic change. This discussion will help clinicians in making correct diagnosis by correlating history, physical examination and US findings and moreover, may help avoiding in-appropriate anticoagulation e.g., direct oral anticoagulant dose loading when DVT is chronic or when no longer required e.g., chronic DVT from major transient risk factors [8, 9]. We discussed CDUS findings with the reporting radiologist and concluded the findings were indeterminate on CDUS and attributed his distal DVT as provoked, secondary to an ICU stay 10 weeks ago. The recommended anticoagulation treatment duration for provoked distal DVT in updated in CHEST 2021 guidelines is 12 weeks [10]. On the other hand, THANZ (Thrombosis and Haemostasis Society of Australia and New Zealand) 2019 VTE guidelines proposed anticoagulation treatment duration for provoked and unprovoked distal DVT to be 6 and 12 weeks respectively [8]. We felt that the age of the DVT was close to three-months duration based on the reported history and decided not to treat with anticoagulation. We suggested use of compression stockings in view of CVI and ceased further ultrasound monitoring.

### Case 2: Residual venous occlusion (RVO) or partial thrombus on CDUS.

A 50-year-old gentleman presented with acute left leg swelling, pain and erythema suggestive of acute cellulitis. He had past history of unprovoked left popliteal and superficial vein thrombosis one year previously, however rivaroxaban was discontinued after six months of therapy as the patient was not keen to continue. CDUS was arranged in view of previous history of DVT, which showed a partial thrombus in the superficial femoral and popliteal vein with no extension of thrombus to other parts of the deep venous circulation. There was partial compressibility of the echogenic thrombus within the lumen of the veins and peripheral revascularization with evidence of collateralization.

Was US indicated in this patient, despite a clear cellulitis diagnosis?

Should we consider anti-coagulation extension or discontinuation based on the presence or absence of residual thrombus?

#### Discussion

This patient had clinical features suggestive of acute cellulitis. We believe, that repeating the US in this patient was appropriate as this patient had no end of anticoagulation therapy US when he previously decided to stop anticoagulation for unprovoked DVT, which carries high risk of future VTE recurrence.

Routine use of venous US among patients with a clear cause of acute limb swelling e.g. cellulitis with past history of DVT appears to be a grey area which requires further studies to determine who will benefit from US assessment. Ordinarily, venous US may be considered in those patients where diagnosis of cellulitis is in question or the patient doesn’t have any evolving skin and soft tissue changes suggestive of cellulitis and other non-infectious mimickers of cellulitis appear unlikely. DVT occasionally presents with erythema which mimics cellulitis.

Partial thrombus finding on ultrasound is often seen when it is repeated several months following acute DVT. Residual venous occlusion (RVO) is another nomenclature used to describe the residual thrombus. Data over the past two decades has shown discordant association between RVO and recurrent DVT. RVO has long been considered as a pro-thrombotic state, which may predict subsequent risk of DVT recurrence [11]. RVO has impacted clinical practice and resulted in routine US monitoring to extend or stop anticoagulation based on the status of venous thrombus. A few systematic reviews have been undertaken, which are mainly based on observational studies [12–16], and 1 randomized trial by Prandoni et al. [17] evaluated the use of ultrasound assessment for residual thrombosis from an initial DVT to guide the duration of anticoagulation.

They concluded that tailoring the duration of anticoagulation based on the ultrasound findings reduced the rate of proximal DVT recurrence with a non-significant reduction in the risk of VTE. However, the investigators reported several methodological limitations such as lack of double blind design, underpowered sample size, and the exclusion of patients with previous VTE, permanent VTE risk factors and inherited thrombophilia.

Conversely, there are studies questioning the value of RVO as predictor of VTE recurrence. Le Gal et al. [18], Carrier M [12] and Cosmi B [19] did not find any association between RVO and VTE risk recurrence.

It should be noted that resolution of DVT in anticoagulated patients is slow [20, 21].

Society of Radiologists in their latest consensus report provide an alternative interpretation of chronic partial or residual thrombus seen on interval US. After DVT, the vein may heal completely or scar. Thrombus undergoes a series of changes over the weeks to months following a DVT, which may culminate in development of fibrosis resulting in vessel wall thickening, scarring and synechiae causing partial obstruction, otherwise known as vein wall remodelling [22]. The residual material is no longer considered a thrombus [6].

It appears that persistence of thrombus beyond three months of adequate anticoagulation does not mean treatment failure or warrant extension of anticoagulation unless significant risk of VTE recurrence exists [8, 9, 23, 24]. We attributed his residual left leg swelling as mild post-thrombotic syndrome and interpreted the US findings as chronic scar or post-thrombotic change, which doesn’t represent a manifestation of recurrent DVT.

### Case 3: Interval increase in thrombus size or complete thrombosis of one or more deep veins on follow-up US.

A 59 year Chinese gentleman was admitted after head injury from a recent fall. CT brain showed acute on chronic sub-dural hematoma (SDH). The patient was receiving oral anticoagulation with rivaroxaban for a left unprovoked popliteal vein and common femoral vein DVT, which had been diagnosed nine months before. Upon admission, US was repeated which showed interval progression to complete thrombosis of the common femoral vein and partial thrombosis of the popliteal vein. Patient denied any worsening of the left leg swelling since the diagnosis of DVT nor any new leg symptoms. US was interpreted as acute, recurrent left proximal DVT by the treating physician and an IVC filter was ordered to mitigate DVT risk as per neurosurgical advice. Anticoagulation was held-off and burr hole surgery to evacuate the SDH was planned.

**How should we interpret US findings and does this patient has recurrent DVT?**

#### Discussion

Occasionally, US when repeated for proximal DVT patients, either as follow-up or due to persistent leg symptoms shows a finding of interval complete thrombus occlusion in a previously partially occluded venous segment or extension of thrombus to a new segment (propagation) within the same previously affected vein. This ultrasound report might be interpreted as DVT recurrence among asymptomatic patients (without new signs and symptoms since initial DVT diagnosis) and might raise the questions of whether anticoagulation failed while on anticoagulation (i.e., breakthrough DVT) and ultimately may lead to change in type of anticoagulation or resumption of anticoagulation if previously stopped.

An older study by Mark H et al. [25] noted that re-thrombosis events were common after an acute DVT (30% of initially affected extremities), of which propagation of thrombus was an early event occurring within a median of 40 days, while re-thrombosis was a late event. The aim of this study was to evaluate the impact of thrombotic events on venous valve function and concluded that such thrombotic events are unrelated to recognized clinical risk factors and may occur despite standard anticoagulation. The study did not record clinically relevant thrombo-embolic outcomes. Another older study by Krupski et al. [26]observed thrombus propagation among one third of nine adequately anticoagulated patients and concluded that these events were not necessarily synonymous with anticoagulation treatment failure. The true clinical significance of thrombus propagation and/or increase in thrombus diameter remains unclear. It has been postulated that an increase in thrombosis in a partially occluded vein within the first few weeks after diagnosis may, in part, reflect progressive occlusion of the vein lumen caused by increased thrombus adherence and retraction [27]. The propagation of thrombus noted within the first few weeks since diagnosis may represent less extensive involvement initially, reflecting the earlier presentation of these patients [25].

We understand that diagnosing a new DVT after a prior DVT might be challenging. Thrombus propagation or complete occlusion of a previously partially occluded vein on follow-up US in the absence of any new DVT symptoms, should not be considered a case of DVT recurrence, as there is no supportive evidence that sub-clinical propagation of thrombus in adequately anticoagulated patients influences clinical outcomes.

In our opinion, repeating US without any new leg symptoms was questionable. The patient had remained compliant to anticoagulation throughout the past nine months. As explained in the discussion above, complete occlusion or partial thrombosis on an interval US without any new compatible leg DVT symptoms, is likely to represent chronic post-thrombotic change. In-view of the acute SDH, we recommended intermittent pneumatic compression as DVT prophylaxis and recommended against the use of IVC filter, which may be considered in the acute phase of a proximal DVT of < 3 months. Anti-coagulation was resumed post-surgery as per neuro-surgery recommendations.

### Case 4: Persistent leg swelling and pain in DVT limb following recent DVT.

A 35-year-old Malay gentleman without any significant past medical history presented with an unprovoked right sided extensive proximal DVT.

Long-term anticoagulation was recommended without a stop date. At six-months follow-up, he complained of improved but persistent leg swelling and pulling calf pain since the onset of DVT but he denied calf tenderness.

Should we repeat an US in-view of his persistent leg swelling despite anti-coagulation or continue current anticoagulation?

#### Discussion

A significant number of patients with DVT continue to experience persistent leg swelling and pain despite receiving anticoagulation. These patients are at risk of post-thrombotic syndrome (PTS) [8]. There is no evidence that extension of anticoagulation reduces severity or future risk of PTS [8]. Persistent but significantly improved leg swelling should not be the reason to repeat US, with a view to find a new thrombus or RVO. However, as mentioned above, US may be performed if confirmation of PTS would facilitate further management such as endovascular intervention or unless a change in management is expected. In this context, anticoagulation may continue if the patient has an unprovoked DVT or has minor transient or major persistent risk factors [8, 9, 23, 24, 33] for extended anticoagulation (low bleeding risk).

This patient most likely had PTS, we recommended long-term anticoagulation without a stop date in view of the unprovoked DVT and did not perform any repeat US. PTS is considered a risk factor for DVT recurrence, however from guidelines it appears that the factors favouring long-term anticoagulation appear to be VTE recurrence risk from VTE risk factors (transient and persistent) rather than presence of residual leg swelling or PTS per se [6, 8, 23, 24, 28, 29, 33].

### Case 5: Provoked distal DVT, not treated with anticoagulation.

A 50-year-old Chinese lady with poor mobility secondary to recent fall which resulted in right big toe fracture, was noted to have right lower limb swelling. CDUS showed distal DVT involving the right posterior tibial vein which was 3.8 cm in length. In view of her high bleeding risk contributed the high falls risk, chronic kidney disease and new onset anaemia, a decision was made to follow the US surveillance route rather than to anti-coagulate.

What are the suggested US surveillance schedule and indications to start anticoagulation based on repeat US findings?

#### Discussion

Guidance on surveillance US schedule has been addressed in the SRU consensus report [6] and in the recently updated CHEST guidelines [10]. The recommendation is to perform serial ultrasounds once weekly for a total of 2 weeks. However, US may be repeated earlier than the recommended schedule in any case of worsening symptoms. If calf thrombus propagation is noted at any US, anticoagulation may be initiated in low bleeding risk patients and further ultrasound surveillance is ceased.

If distal thrombus remains stable during surveillance, further scanning after 2 weeks is generally not warranted, as this indicates a lower risk of subsequent thrombus extension.

CDUS was arranged after 1 week, which showed increase in thrombus length to 7.7 cm without any change in calf signs and symptoms. Due to the high bleeding risk, anticoagulation was not initiated and a decision was made to repeat CDUS 1 one week later with close monitoring of patient’s leg signs and symptoms. The repeat CDUS showed a reduction in thrombus size to 1.5 cm.

Due to paucity of high quality evidence, uncertainty remains as to which patients would benefit from anticoagulation or ultrasound surveillance.

The highest risk of extension is usually within the first week of DVT onset [20]. Due to paucity of high quality data on distal DVT, it remains unclear if any increase in thrombus size within the same venous segment without any worsening in clinical features of DVT at follow-up US represents worsening of DVT? For this scenario above, a weak recommendation has been proposed to treat with anticoagulation in the recently updated CHEST guidelines [10]. However, extension to a femoro-popliteal vein would certainly warrant initiation of anti-coagulation, provided the risk of bleeding is deemed low.

We ceased further US surveillance and instructed the patient to monitor for any new symptoms indicating recurrence of DVT. We also informed the patient that the long-term risk of recurrence after a first provoked distal DVT is significantly lower than a proximal DVT.

## Section II. A suggested guidance on appropriate venous ultrasound use in long-term lower extremity DVT follow-up

The SRU 2018 consensus statement describes appropriate frequency of follow-up ultrasound after initial negative and positive US, including indeterminate or equivocal US.

In-order to avoid duplication, we will not discuss these further.

However, neither the SRU document nor any other guide, provides a clear guidance on role of venous US in long-term DVT follow-up. Here, we present our Suggest to based on best available evidence on follow-up US according to VTE type and VTE risk factors, which we hope may be helpful to clinicians who routinely follow-up patient with VTE. (Table 1)

In order to understand role of US in longitudinal DVT follow-up, it is important to discuss various international guideline recommendations on issues of RVO and role of end of therapy US.

### Role of end of anticoagulation therapy US

Guidelines have described purpose of US at end of anticoagulation therapy to establish a new baseline for future comparison: This will help in differentiating which veins have recanalized and which ones are scarred.

European society for vascular surgery (ESVS) 2021 (Class IIb, level C) [23], THANZ 2019(Grade: Strong; Evidence: low) and SRU 2018 suggest consideration of venous US at the end of anticoagulation treatment. These guidelines do not describe whether DVT requiring 3 months of treatment i.e., provoked and distal would benefit similarly to unprovoked DVT.

NICE 2020 VTE [29] guidelines describe extension of anticoagulation based on risk of VTE recurrence (unprovoked or provoked risk factors) and similar to updated CHEST 2021 [10] and American Society of Hematology (ASH) 2020 VTE guidelines [24] do not describe the role of end of therapy US in DVT follow-up.

We agree with Meissner’s review [4] that patients who may benefit most from end of therapy US are those who are at high risk of future DVT recurrence, as it may be difficult to differentiate acute recurrent thrombus from prior scarring.

We are of the view that scarring develops over a period of time and repeating US at end of 3-months of anticoagulation e.g., provoked DVT is unlikely to be helpful due to the low risk of future DVT recurrence and scarring is unlikely to develop around this time.

### Anticoagulation decision making based on status of interval venous occlusion on repeat US

Equipoise sometimes exists after the first unprovoked VTE as patients often query if they can stop anticoagulation after a period of 3–6 months. NICE 2020, THANZ 2019 and CHEST 2021 updated guidelines do not describe a prognostic role of RVO in VTE recurrence.

THANZ 2019, ASH 2020 and SRU 2018 guidelines [6, 8, 24] recommend against routine use of US to detect RVO to guide the duration of anticoagulation among patients with unprovoked VTE. Despite recommendation against the routine use of US, ASH 2020 (conditional recommendation) and ESVS 2021 (class IIb, level B) [23] issued recommendation that under certain circumstances, when risk benefit ratio is uncertain to continue anticoagulation following DVT US for residual thrombus may aid patients in making a final decision.

Duration of anticoagulation decision making based on presence or absence of RVO remains an unresolved matter due to lack of consensus among various guidelines. However, it appears that most guidelines tend to suggest anticoagulation based on long-term VTE recurrence, bleeding risk and patient preference rather than repeating US to detect presence or absence of residual thrombus.

### Future potential indication of follow-up US

US assessment of Residual Vein Thrombus (RVT) in patients with proximal DVT has the potential to identify those who are likely to benefit from long-term use of Elastic Compression Stockings (ECS). RVT and or popliteal valve reflux (PVR) at 6 weeks following proximal DVT on US in a recent meta-analysis was associated with higher risk of future PTS [30].

A recent sub-analysis [31] of a previously published study [32], suggested that adequate use of ECS in proximal DVT provides clinically important reduction in any and severe PTS, in patients with US evidence of RVT with or without PVR at 3 months.

Though, the study results appear promising, we await further guidance from major clinical guidelines before adopting these findings into clinical practice.

**Table 1:** A suggested guidance on appropriate venous US use according to type and risk factors in long-term follow-up of lower extremity DVT

Type of DVT	Timing of Venous US
**P**roximal DVT provoked from major transient risk factors Or Distal DVT ( provoked or unprovoked)	End of anticoagulation therapy ultrasound is less likely to be beneficial as treatment duration is time limited, i.e., 3 months and risk of future recurrence low [8,9, 23, 24, 33].
**P**roximal DVT provoked from minor transient risk factors e.g.,air travel	Guidance on duration of anticoagulation beyond three months in this scenario differs among Various guidelines. Some suggest extended anticoagulation indefinitely [9]. Others suggest extesion only after evaluation of thrombotic and bleeding risk with periodic evluation and patient preference [8, 23]. A follow-up ultrasound at the end of planned anticoagulation may be considered.
**P**roximal unprovoked DVT or Proximal DVT provoked from major persistent risk factors e.g., active cancer.	Clinical “equipoise” is common regarding extension of anticoagulation beyond 3-6 months. after first unprovoked VTE. Most guidelines suggest indefinite anticoagulation among low bleeding risk patients [9, 24] or extended anticoagulation based on patient preference [8] and periodic bleeding re-assessment [23]. Among cancer assoicated thrombosis, patients with low bleeding risk would benefit from extended anticogulation as long as cancer ramains active [8, 9, 23, 24]. CDUS should be considerd in event of suspected DVT recurrence and may be considered towards end of planned anticoagulation. .
**D**istal provoked or unprovoked DVT, not treated with anticoagulation	Serial ultrasound once weekly, or earlier if worsening symptoms, for 2weeks Further scanning beyond 2 weeks is generally not warranted [6, 10].
**R**esidual venous occlusion or partial thrombus on end of therapy US follow-up	Further ultrasounds are unlikley to be beneficial as most guidelines support decision to continue or stop anticoagulation based on the risk of VTE recurrence from unprovoked or provoking risk factors, bleeding risk and patient preference. [6, 8, 24, 29].

## Summary

- We recommend venous US reporting in health systems to be standardized in line with the Society of Radiologists in Ultrasound consensus statement as acute, indeterminate and post-thrombotic change to allow treating physicians to make appropriate decisions.
- End of therapy US appears appropriate among patients who are candidates for long-term or infinite duration of anticoagulation, should they decide to stop anticoagulation.
- Most guidelines suggest duration of anticoagulation based on recurrence risk form underlying VTE risk factors (transient and persistent). However, significance of RVO in determining duration of anticoagulation remains a grey area.
- Given the lack of consensus by published guidelines, we recognize the need for higher quality studies, including that of randomized control trials to evaluate the role of lower limb CDUS as part of the management strategy following a prior diagnosis of lower limb DVT, as well as the analysis of real-world data from large VTE registries and databases.

## Data Availability

N.A.

## List of abbreviations

DVT: Deep vein thrombosis
CDUS: Complete duplex ultrasound
PTS: Post-thrombotic syndrome
THANZ: (Thrombosis and hemostasis society of Australia and New Zealand)
NICE: National institute of clinical excellence
ESVS: European society for vascular surgery
SRU: Society of Radiologist in ultrasound
ASH: American society of hematology
SDH: Sub-dural hematoma
IVC: Inferior vena cava
